# An Exploratory Study on the Antibiotic Potentiation Activity of Riboflavin Against Biofilm‐Forming Multidrug‐Resistant *Salmonella* and *Shigella* Clinical Isolates

**DOI:** 10.1155/ijm/1772416

**Published:** 2026-10-29

**Authors:** Abiola Isawumi, Gloria Kutorkor Addo, Elizabeth W. Bugase, Eunice Ampadubea Ayerakwa, Molly Kukua Abban

**Affiliations:** ^1^ West African Centre for Cell Biology of Infectious Pathogens Department of Biochemistry Cell and Molecular Biology University of Ghana Accra Ghana ug.edu.gh; ^2^ AMR Research Group West African Centre for Cell Biology of Infectious Pathogens College of Basic and Applied Science University of Ghana Accra Ghana ug.edu.gh

**Keywords:** 3D chequerboard, antibiotic adjuvant, biofilms, fractional inhibitory concentration, meropenem synergy, riboflavin

## Abstract

*Shigella* and *Salmonella* strains have been implicated in neonatal sepsis, food poisoning, and bacteremia. Their increasing resistance to antibiotics has undermined treatment options, constituting a public health crisis. In addition, the scarcity of new antibiotics contributes to multidrug‐resistance (MDR). Hence, there is a need to potentiate antibiotics to mitigate MDR. This study assessed riboflavin′s activity to potentiate antibiotics against biofilm‐forming MDR *Shigella* and *Salmonella* spp. Disk diffusion, microbroth‐dilution, chequerboard, and time‐kill assays were used to determine resistance profiles and riboflavin‐antibiotic activity. Biofilm‐formation and bacterial viability (after riboflavin–antibiotic challenge) were determined with crystal‐violet and macrophage infection assays, respectively. Strains exhibited > 80% resistance to fluoroquinolones and carbapenems, with 160–640 *μ*g/mL MIC ranges. At 40–80 *μ*g/mL riboflavin, there was a twofold reduction in bacterial survival, with meropenem–riboflavin exhibiting significant reduction. Meropenem + riboflavin was highly synergistic in four MDR isolates (an FIC of 0.075). Biofilm formation was altered (from strong to weak phenotype) with 40 *μ*g/mL riboflavin–antibiotic treatment. There was a 40%–70% reduction in bacterial survival in THP‐1 cells treated with meropenem–riboflavin after 3 h, and complete clearance between 24 and 48 hpi. Overall, the study showed that riboflavin potentiated fluoroquinolones, meropenem, and enhanced clearance in THP‐1 assays.

## 1. Introduction

*Shigella* and *Salmonella* spp. are global priority pathogens with a consequent public health crisis [1]. They cause severe infections including food poisoning, salmonellosis/shigellosis, bacteremia, and neonatal sepsis [2]. These infections are difficult to treat as bacteria develop resistance to antibiotics and form biofilms [3], thereby contributing to the rising challenges of antimicrobial resistance (AMR). This is compounded by the dearth of novel antibiotics in the development pipeline, necessitating the need to potentiate or repurpose existing antibiotics to enhance effectiveness. Small molecules such as HSD1624, SG‐B‐52, and SMA (small molecular adjuvant) have been explored as adjuvants to enhance the efficacy of antibiotics and demonstrated antibiofilm properties [4, 5, 6].

Riboflavin, a promising small molecule has been indicated to enhance antibiotic activity by inhibiting bacterial growth [7]. It contributes to the function of innate immune systems as microbiological barriers and a first line of protection against pathogens [8]. It achieves this by triggering macrophage phagocytosis and suppressing the production of proinflammatory cytokines (IFN‐*γ*, IL‐6, and TNF‐*α*) [8, 9]. These cytokines are expressed in sepsis, especially in association with multidrug‐resistant and biofilm‐producing bacteria such as *Shigella* and *Salmonella* spp. [10, 11]. Additionally, riboflavin in combination with antibiotics such as ciprofloxacin and azithromycin reduces bacterial infection [8].

In Ghana, our recent study isolated multidrug‐resistant *Shigella* and *Salmonella* implicated in neonate sepsis from hospital intensive care units (ICUs). These bacterial strains were resistant to frontline conventional and last‐resort antibiotics including fluoroquinolones and carbapenems. This study, therefore, explored riboflavin to potentiate fluoroquinolones and meropenem against biofilm‐forming MDR *Shigella* and *Salmonella* isolates.

## 2. Materials and Methods

### 2.1. Strain Information

Four archived *Salmonella enterica* (SAL901, SALAB) and *Shigella flexneri* (SH701, SHAB) strains from ABISA Bacterial Culture Library at West African Centre for Cell Biology of Infectious Pathogens (WACCBIP), University of Ghana were used in this study with approval from Ghana Health Service IRB (GHS‐ERC01/02/19). The strains were isolated from neonatal sepsis (as guided by GHS and participants′ informed consent) and are MDR to fluoroquinolones, carbapenems, *β*‐lactams, penicillins, macrolides, chloramphenicol, sulfonamides, and cephalosporins. All the strains were revived on MacConkey agar media (37°C for 24 h) (Oxoid, England, CM0003) prior to the experiments. The growth rate of the strains was determined with a standard growth curve assay [12], and a preprint has previously been published [13]. Assay performance was verified with ATCC25922 (QC). ATCC25922 is a QC organism and not a species‐matched baseline; resistance framing was aligned to CLSI species‐specific breakpoints. All assays were conducted in two independent experiments with three replicates.

### 2.2. AMR Profiling Assays

Kirby–Bauer disk diffusion was used to determine AMR profiles of the strains to ciprofloxacin (10 *μ*g/mL), levofloxacin (10 *μ*g/mL), ofloxacin (10 *μ*g/mL), and meropenem (10 *μ*g/mL) (Mast Diagnostics, Mast Group Ltd., Merseyside, United Kingdom) as previously described [12]. Sterile Mueller–Hinton agar plates (Oxoid, Basingtoke, United Kingdom) were inoculated with standardized overnight culture (0.5 at 600 nm, GENESYS 10S UV‐VIS) of the strains and antibiotic disks applied aseptically and incubated (37°C for 24 h). The zone of inhibition determined using CLSI guidelines in two independent experiments with three replicates. The ratio of antibiotics resisted by test strains to the total number of antibiotics tested expressed in percentages was used to determine percentage of resistance. To determine the minimum inhibitory concentrations (MIC), 100 *μ*L of the standardized culture (Mueller–Hinton broth) was added to 100 *μ*L of the tested antibiotics in a 96‐well plate and incubated (37°C for 24 h, 120 rpm). The OD (600 nm) was measured, and ratio of the OD of the test strain to the control expressed in percentages was used to determine the MIC. The percentage OD < 10 was the assigned MIC of the strains at the indicated concentrations. The fractional resistance was achieved in a colony‐spotting assay by comparing the percentage of colonies from antibiotic‐resistant wells to the control without antibiotics as previously described [12].

### 2.3. Riboflavin Challenge and Chequerboard Assays

A cocktail of riboflavin and test antibiotic was prepared to reduce AMR levels of the strains. Briefly, 50 *μ*L of the standardized concentrations of the antibiotics (10 *μ*g/mL—meropenem, ofloxacin, ciprofloxacin, and levofloxacin) was combined with 50 *μ*L of experimental concentrations of riboflavin (10, 20, 40, and 80 *μ*g/mL) in 96‐well plates. Approximately 100 *μ*L of the standardized culture of the strains was added to the cocktail, incubated (37°C for 24 h) and optical density determined (600 nm). To determine the in vitro synergistic activity of riboflavin and the tested antibiotics (meropenem, ofloxacin, ciprofloxacin, and levofloxacin) against MDR *S. flexneri* and *S. enterica*, a checkerboard experiment was conducted.

Firstly, the effects of the test antibiotics at their standard concentrations (10 *μ*g/mL) in combination without the riboflavin were determined. Briefly, the antibiotics were prepared in twofold increasing concentrations of (10, 20, 40, 80, 160, 320, 640, and 1240 *μ*g/mL). A total of 50 *μ*L of MHB was transferred into each well of microtiter plates and first antibiotic was serially diluted along the ordinate (96‐well plate *y*‐coordinate), whereas the second along the abscissa (96‐well plate *x*‐axis) as previously described [14]. The microtiter wells were inoculated with 50–100 *μ*L of the standardized inoculum (0.5 at 600 nm) of the strains and incubated (37°C for 24 h, 120 rpm) under aerobic conditions. The MICs were determined as previously described, whereas fractional inhibitory concentrations index (FICI) was calculated as FICI = [(MIC Antibiotic X in combination/MIC Antibiotic X) + (MIC Antibiotic Y in combination/MIC Antibiotic Y)] [14]. Synergistic activity was defined as FICI ≤ 0.5, no interaction (0.5 < FICI ≤ 4) and antagonistic (FICI > 4). The same experimental procedure was repeated to determine the effects of riboflavin in combinations with the antibiotics, however, with varying and different concentrations per assay. For riboflavin with meropenem, double concentrations of both were used in increasing orders (1, 2, 4, 8, 16, 32, 64, and 128 *μ*g/mL), and FICI determined as previously indicated. Since meropenem is a last‐resort antibiotic, the effects of riboflavin on combinations of meropenem and other test antibiotics (ciprofloxacin, levofloxacin, and ofloxacin) were determined in a three‐dimensional chequerboard assay as previously described [15]. Similar to the previously described procedure, 40 *μ*g/mL of riboflavin was added to twofold increasing concentrations of ciprofloxacin, levofloxacin, and ofloxacin (10, 20, 40, 80, 160, 320, 640, and 1240 *μ*g/mL) in the 96‐well plates with 100 *μ*L of standardized inoculum and incubated at already stated conditions and interpreted as previously described. All the assays were conducted in two independent experiments with three replicates.

### 2.4. Riboflavin–Antibiotic Biofilm and Time‐Kill Curve Assays

Crystal violet biofilm assay was conducted as previously described [16, 17]. Bacterial cultures were prepared in 96‐well microtiter plates containing minimal media supplemented with glucose. The plates were incubated for 48–72 h at 37°C; the planktonic cells (nonadherent cells) were removed with 0.9% normal saline (two to three times) and washed gently with sterile ultrapure water. Approximately 200 *μ*L of 0.1% crystal violet was added to each well and incubated at room temperature for 30 min. The crystal violet (solubilized with 96% ethanol or glacial acetic acid) was transferred into another fresh microtiter plate and OD was measured (590 nm). The experiment was conducted in triplicate. Biofilm biomass was calculated by first establishing OD cutoff value (ODc) as previously described [18]. ODc is average OD of negative control +3 × standard deviation (SD) of negative control. OD of the tested strain is average OD of strain reduced by ODc value. The results were interpreted as non (OD ≤ ODc) or weak (ODc < OD ≤ 2 × ODc) or moderate (2 × ODc < OD ≤ 4 × ODc) or strong (4 × ODc < OD) biofilm formation.

To determine the effects of riboflavin on biofilm formation of the strains, established MIC of the strains were used as baseline for biofilm inhibition or eradication assay. A total of 100 *μ*L of the standardized culture (OD–0.5) was transferred into 96‐well plates seeded with various concentrations of antibiotics: 160–320 ug/mL of the antibiotics for the *Salmonella* strains, 320–640 ug/mL for the *Shigella* strains, incubated (37°C, 48–72 h), and biofilm formation was determined as previously indicated and products quantified with resazurin dyes. Time‐kill activity of the riboflavin–antibiotic cocktail was performed in triplicate according to the microbroth dilution protocol guided by CLSI criteria and as previously described [19]. As already indicated, 500 *μ*L of (10, 20, 40, and 80 *μ*g/mL) riboflavin–antibiotic (10 *μ*g/mL of each antibiotic) cocktail prepared in 1:1 was added to 500 *μ*L of the strains at the log phase. The cultured strains were normalized to MacFarland standard and 1 mL of the inoculum with 1 mL of the prepared cocktail in test‐tubes were incubated at 37°C for 24 h with shaking (125 rpm). To determine the time‐kill activity in bacterial cells/mL, 30–50 *μ*L was withdrawn at 1 h intervals for 28 h, spread on LB plates and incubated as earlier stated. Bactericidal activity will be defined as ≥ 3 log10 CFU/mL reduction and synergy in time‐kill will require ≥ 2 log10 CFU/mL.

### 2.5. Infection and Bacterial Survival Index Assays

The survival index of the strains was determined using THP‐1 cell line cultured in DMEM supplemented with penicillin and streptomycin (50–100 *μ*g/mL) and 10% FBS as previously described [10, 11]. Briefly, the macrophage cell was initially seeded at a 1 × 10^6^ cells/mL for 24–48 h for adherence. The bacterial cultures were standardized to 1 × 10^6^–1 × 10^8^ cells/mL and cocultured with the cell line. Antibiotics (10 *μ*g/mL ciprofloxacin and 10 *μ*g/mL meropenem) and riboflavin (10, 20, 40, and 80 *μ*g/mL) were added and incubated (24–48 h, 37°C in 5% CO_2_) with controls (untreated cell line). Viability was determined with 10% (v/v) resazurin and incubated as previously indicated. Absorbance was determined at 570 nm, and bacterial survival was estimated and expressed in percentage relative to the control.

## 3. Results

### 3.1. Growth Profile and AMR Profile of Bacterial Strains

Resistance in *Salmonella* and *Shigella* strains was interpreted against CLSI species‐specific breakpoints. *Salmonella* and *Shigella* strains were resistant by MIC values and CLSI/EUCAST criteria, and the data are presented in zone diameters and categorical calls (Figure 1A and Table 1A). The highest level of resistance (90%–95%) was observed in the presence of levofloxacin, followed by ofloxacin (90%–92%), meropenem (80%–85%), and ciprofloxacin (81%–85%). Growth rate increased with increase in incubation time for all strains (Figure 1B). Standard growth profile indicated that strains grew appreciably well at 37°C with initial lag phase of 2 h. Relative to ATCC25922 which displayed exponential growth over 18 h indicating robust and actively dividing bacterial cells at 37°C.

**Figure 1:**
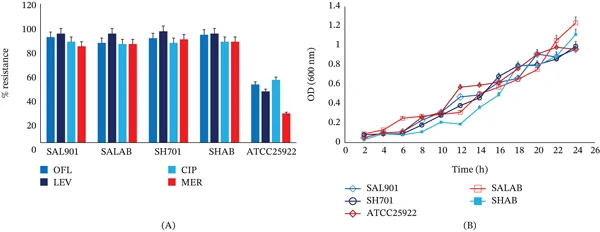
Percentage antimicrobial resistance and growth profile of *Salmonella* and *Shigella* strains. (A) Resistance profile of strains in the presence of fluoroquinolones (ofloxacin, levofloxacin, and ciprofloxacin) and meropenem at standard antibiotic concentrations. OFL, ofloxacin; LEV, levofloxacin; CIP, ciprofloxacin; MER, meropenem. (B) Growth profile for Salmonella strains (SAL901 and SALAB) and Shigella strains (SH701 and SHAB) at 37℃; absorbance at 600?nm plotted against time, h

**Table 1:** Minimum and fractional inhibitory concentrations and biofilms profiles.

Strain	A. Minimum inhibitory concentration (*μ*g/mL)									
	OFL	LEV	CIP	MER	OFL + LEV	OFL + CIP	LEV + CIP	OFL + MER	LEV + MER	CIP + MER
SAL901	320	320	320	160	320	320	320	160	160	160
SALAB	320	320	160	160	320	320	320	160	160	160
SH701	640	320	640	320	320	320	320	160	160	320
SHAB	640	320	320	640	320	320	320	160	320	160
		

**Strain**	**>B. Fractional inhibitory concentration**									
	**OFL + LEV**	**OFL + CIP**		**LEV + CIP**		**OFL + MER**		**LEV + MER**		**CIP + MER**

SAL901	2.0	2.0		2.0		1.5		1.5		1.5
SALAB	2.0	3.0		3.0		1.5		1.5		2.0
SH701	1.5	1.0		1.5		0.8		1.0		3.0
SHAB	1.5	1.5		2.0		0.5		1.5		0.8


**Strain**		**C. Biofilm profiles: strain + antibiotics (*μ*g/mL)**								
		**OFL (10)**		**OFL + RIB (40)**	**LEV (10)**	**LEV + RIB (40)**	**CIP (10)**	**CIP + RIB (40)**	**MER (10)**	**MER + RIB (40)**

SAL901	Strong	Strong		Weak	Strong	Weak	Strong	Weak	Strong	Weak
SALAB	Strong	Strong		Weak	Strong	Weak	Strong	Weak	Strong	Weak
SH701	Strong	Strong		Weak	Strong	Weak	Strong	Weak	Strong	Weak
SHAB	Strong	Strong		Weak	Strong	Weak	Strong	Weak	Strong	Weak

Abbreviations: CIP, ciprofloxacin; LEV, levofloxacin; MER, meropenem; OFL, ofloxacin.

### 3.2. MIC, FIC, and Biofilm Profiles of Strains

Microbroth dilution and checkerboard assay were used to establish the MIC and FIC of the strains (Table 1A). Individual MIC for *Salmonella* (SAL901, SALAB) ranged between 160–320 *μ*g/mL against tested fluoroquinolones MICs (160–320 *μ*g/mL) exceeded CLSI/EUCAST resistance breakpoints, corroborating categorical resistance across all antibiotics. Combinations of all the test antibiotics exhibited an MIC of 160–320 *μ*g/mL; however, there was a twofold reduction in the MIC for fluoroquinolones when combined with meropenem in some cases. The FIC index value for *Salmonella* (SAL901, SALAB) combinations with fluoroquinolones and meropenem showed no interaction (Table 1B).

For SH701 and SHAB, MIC values ranged between 320–640 *μ*g/mL for individual antibiotics (Table 1A); however, fluoroquinolone combinations with meropenem resulted in a twofold reduction in MIC (160–320 *μ*g/mL). The FIC between 0.5 and 3.0 (Table 1B), indicative of no interaction, showed a slight increase in inhibitory activity when combined (Table 1A).

*Salmonella* and *Shigella* strains were strong biofilm formers with and without standard concentration of antibiotics (Table 1C). When combined with riboflavin at 40 *μ*g/mL, there was a change in biofilm phenotype from strong to weak in all strains (Table 1C).

### 3.3. Riboflavin Challenge Assay

To determine the effect of riboflavin on bacterial strains treated with fluoroquinolones and meropenem, *Salmonella* and *Shigella* strains were challenged with increasing concentrations of riboflavin (twofold increase). There was no reduction across the tested strains with riboflavin‐only controls (10–80 *μ*g/mL) (Figure 2). All strains displayed gradual reduction in bacterial survival with antibiotic and increasing riboflavin concentrations. At 40–80 *μ*g/mL riboflavin, there was significant bacterial growth inhibition with all the tested antibiotics (Figure S1A, S1B, S1C, S1D: Riboflavin and antibiotics combination assay against MDR *Salmonella* and *Shigella* species).

**Figure 2:**
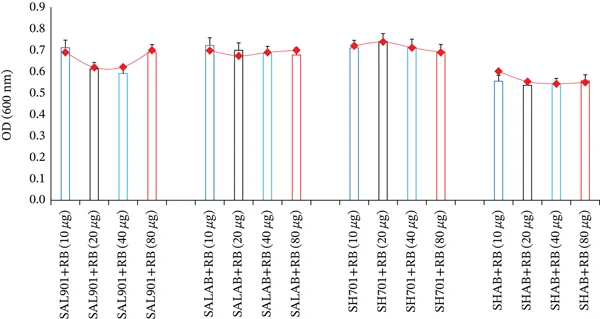
Riboflavin‐only controls showed no reduction in bacterial load across the tested strains.

### 3.4. Meropenem–Riboflavin Challenge

Meropenem–riboflavin challenge exhibited the most significant reduction in bacterial concentration (Figure S1A: Effect of riboflavin and meropenem combination against multidrug‐resistant *Salmonella* and *Shigella* species). At 10 *μ*g/mL riboflavin, survival rate of *Salmonella* and *Shigella* strains was below 10% relative to standard meropenem treatment (10 *μ*g/mL). Survival rate for the strains at the selected riboflavin concentrations (10, 20, 40, and 80 *μ*g/mL) was below 10% indicating significant bacterial inhibition in meropenem‐treated strains challenged with riboflavin.

Because of the reduction in MIC of fluoroquinolones–meropenem combination (from 320 to 160 *μ*g/mL), antibiotic‐treated strains were challenged with riboflavin at 40 *μ*g/mL. In the presence of 40 *μ*g/mL riboflavin, there was significant reduction in bacterial concentration for *Salmonella* strains (Figure 3A). Survival rate was < 10% for the strains challenged with the test antibiotics. For *Shigella* strains, 95% inhibition of bacterial strains was observed relative to the untreated control (Figure 3B). The combination of fluoroquinolones with meropenem in the presence of riboflavin resulted in bacterial reduction. Across two independent experiments (*n* = 2), meropenem–riboflavin combinations produced FIC values at or below the synergy threshold. The observed FIC of 0.075 for meropenem–riboflavin supports a potentiation mechanism (Figure 3C).

**Figure 3:**
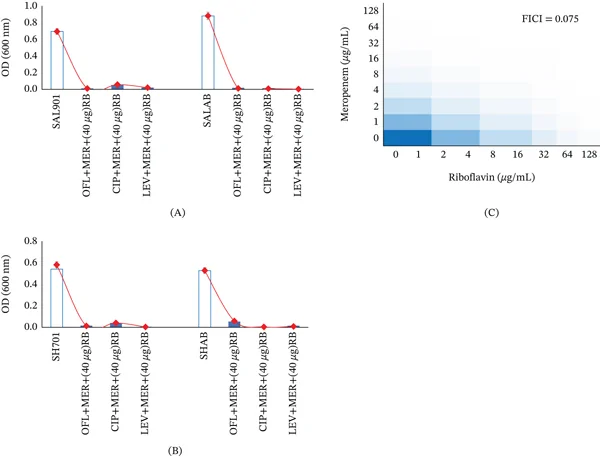
Antibiotic combination of fluoroquinolones and meropenem strains challenged with riboflavin. (A) *Salmonella*‐treated strains challenged with 40 *μ*g/mL riboflavin. (B) *Shigella*‐treated strains challenged with 40 *μ*g/mL riboflavin. (C) FIC of meropenem–riboflavin combination showing synergistic activity. Synergistic activity was defined as FICI ≤ 0.5, consistent with standard checkerboard screening.

### 3.5. Percentage Susceptibility of Individual Antibiotics to Riboflavin

The FIC index value of individual fluoroquinolones challenged with meropenem was above 0.5 with ofloxacin displaying the lowest FIC index of 0.5 and 0.8 for *Shigella* strains, indicative of minimal synergistic activity. When treated strains (fluoroquinolone–meropenem) were challenged with riboflavin, the FIC index values was ≤ 0.5 (Figure 4A), indicative of synergistic activity and evident with the reduction in bacterial concentration following riboflavin challenge (Figure 4). Percentage susceptibility of the strains to riboflavin combination with the antibiotics was > 70% (Figure 4B). Meropenem + riboflavin displayed the highest apparent susceptibility (95%) at 40 *μ*g/mL riboflavin. The level of susceptibility of the strains to fluoroquinolone–riboflavin challenge particularly for ciprofloxacin was > 90% and 70% in *Shigella* with *Salmonella* strains, respectively (Figure 4B).

**Figure 4:**
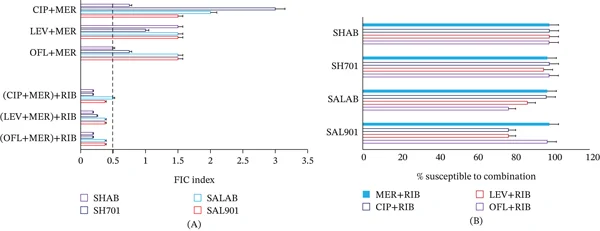
Fractional inhibitory concentration and percentage susceptibility of bacterial strains to antibiotics. (A) FIC index values of fluoroquinolone combination with meropenems and fluoroquinolone–meropenem combination with riboflavin. (B) Percentage susceptibility with riboflavin combination.

### 3.6. Time‐Kill Curve for Riboflavin Challenge

A time‐kill curve assay was performed for strains treated with antibiotics and twofold increasing concentration of riboflavin (10–80 *μ*g/mL) and presented as log10 CFU/mL (Figure 5). Starting with 6 log10 CFU/mL concentration of bacterial cells, 6.7 log10 CFU/mL was observed 16 h after incubation with a gradual increase in CFU/mL over 24 h (Figure 5). Meropenem‐treated strains showed a significant reduction (≥ 50%) within 2 h of riboflavin challenge. Approximately 95% reduction in viable strains was observed 3 h for *Shigella* strains (Figure 5A) at 80 *μ*g/mL riboflavin addition and *Salmonella* strains (Figure 5B) displaying 95% reduction after 5 h challenge. The time‐kill curve for other tested antibiotics including ciprofloxacin, ofloxacin, and levofloxacin was similar to meropenem–riboflavin combination (Figure S2: Time‐kill curve of selected antibiotics in combination with riboflavin at different concentrations).

**Figure 5:**
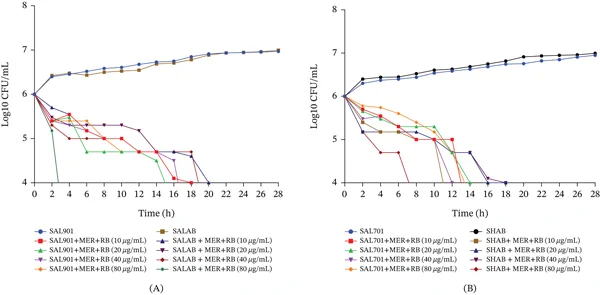
Time‐kill curves for bacterial strains challenged with riboflavin. (A) Log CFU/mL for meropenem–riboflavin challenge against *Shigella* spp. (B) Log CFU/mL for meropenem–riboflavin against *Salmonella* spp.

Addition of increasing concentration of riboflavin to levofloxacin‐treated strains resulted in 10%–25% reduction in log CFU/mL after 2 h. A reduction from 6 log10 CFU/mL concentration to 4 log10 CFU/mL was observed between 6 and 10 h incubation at 10–80 *μ*g/mL riboflavin treatment (Figure S2A: Time‐kill curve of levofloxacin in combination with riboflavin at different concentrations). Ofloxacin‐treated strains resulted in > 90% reduction in CFU/mL. Approximately 15%–20% reduction was observed within the first 2 h of incubation, with gradual decrease in cell concentration (> 1 log10 CFU/mL) between 10 h and 18 h (Figure S2B: Time‐kill curve of ofloxacin in combination with riboflavin at different concentrations). Treatment with ciprofloxacin and riboflavin resulted in threefold to fivefold reduction in log CFU/mL at 2 h. With increased incubation time, ≥ 95% reduction was observed between 5 and 8 h incubation (Figure S2C: Time‐kill curve of ciprofloxacin in combination with riboflavin at different concentrations).

### 3.7. Infection Assay

Bacterial viability after intracellular infection in THP‐1 cells was examined for meropenem and ciprofloxacin‐treated strains. Meropenem (10 *μ*g/mL)‐treated *Salmonella* strains exhibited 90%–100% recovery on plate following macrophage lysis (Figure 6A). For *Salmonella* strains, at 10 *μ*g/mL riboflavin treatment, 90% bacterial load (% CFU/mL) was recovered after 6 h. There was a 40%–70% reduction in bacterial load in macrophages 3 hpi for meropenem strains challenged with 20–80 *μ*g/mL riboflavin. In addition, there was 90% bacterial reduction with meropenem‐treated strains at 80 *μ*g riboflavin after 6 h with complete reduction at 24 h (Figure 6A). For *Shigella* strains, there was 100% bacterial recovery for 10 *μ*g/mL meropenem‐treated strains at 3, 6, 12, 24, and 48 hpi (Figure 6B). Addition of 10 *μ*g/mL riboflavin resulted in 10% reduction in viable bacteria at the different time points. At 20 *μ*g/mL riboflavin treatment, 60%–80% of bacterial concentration was recovered at 24 h postinfection with a twofold reduction at 48 h. Treatment with 40–80 *μ*g/mL of riboflavin resulted in significant reduction (60%) at 3 h postinfection with 90% reduction at 6 h; total inhibition was observed at 24–48 h incubation.

**Figure 6:**
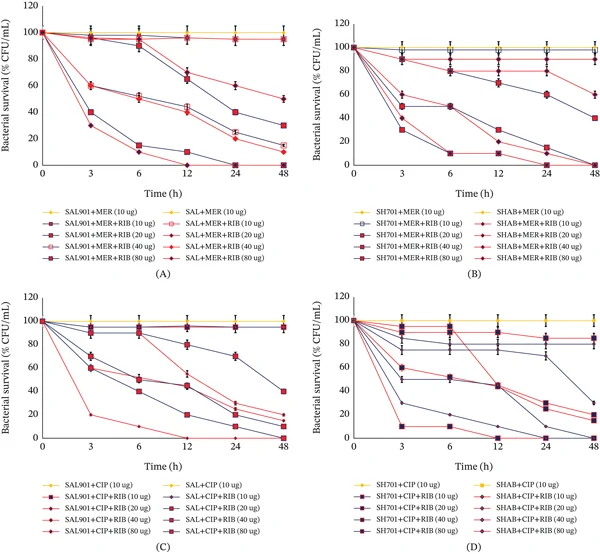
Survival curves for bacterial strains after intracellular infection in cell lines. (A) Survival curve for *Salmonella* strains in meropenem–riboflavin infection. (B) Survival curve for *Shigella* in meropenem–riboflavin infection. (C) Survival curve for *Salmonella* (SAL901 and SAL or SALAB) strains in ciprofloxacin–riboflavin infection. (D) Survival curve for *Shigella* strains in ciprofloxacin–riboflavin infection.

For 10 *μ*g/mL ciprofloxacin treatment, 100% bacterial load was recovered at the five time points for both *Salmonella* and *Shigella* strains (Figure 6C,D). Treatment with 10 *μ*g/mL riboflavin resulted in 5% reduction in bacterial load for *Salmonella* strains and 10%–30% reduction in *Salmonella* strains after 3 hpi. With increasing concentration of riboflavin (twofold increase), there was significant reduction in bacterial load (Figure 6C), where 80 *μ*g/mL resulted in 80%–100% reduction in bacterial load with increasing incubation. For *Shigella*‐treated strains, increasing concentration of riboflavin resulted in significant reduction in bacterial load with 85% reduction 12 h postinfection and 100% reduction at 24 h incubation (Figure 6D).

## 4. Discussion

Multidrug‐resistant *Salmonella* and *Shigella* strains cause infections that are difficult to treat. This necessitates the development of new or resensitization of existing antibiotics with adjuvant‐like agents such as riboflavin. Riboflavin has antimicrobial properties that can potentiate antibiotics and modulate host immune responses [8, 9]. Studies have shown that combination of antibiotics is effective against infections relative to monotherapy [20]. Therefore, this study explored riboflavin in combination with fluoroquinolones and carbapenems against biofilm‐forming MDR *Salmonella* and *Shigella* strains from Ghanaian hospital ICUs.

Riboflavin has been reported to display bactericidal activity in the presence or absence of ultraviolet light radiation [23, 24]. However, significant bactericidal activity with antibiotics and riboflavin combination has been indicated [23, 24, 25]. Previous studies have also explored synergistic interaction of *β*‐lactams and aminoglycosides with significant efficacy on bacterial strains. For example, combination of fluoroquinolones with meropenem reduced *P. aeruginosa* bacterial load [22]. Similarly, levofloxacin and ciprofloxacin were effective against nosocomial pneumonia in adult patients [22]. Our findings showed a similar trend with combination of fluoroquinolones and meropenem, however, with a higher MIC than the recommended standard for individual or combined antibiotics.

The FIC index of meropenem against riboflavin showed a high level of synergy with significant reduction in bacterial population. This FIC‐based inference was conducted with a single riboflavin concentration; however, dose‐response synergy mapping was not performed to confirm synergy across doses. Moreover, this study complemented the assertion that riboflavin best exhibits its antimicrobial activity when combined with other antibiotics such as meropenem and fluoroquinolones to induce synergistic bactericidal activity [8, 26].

In this study, all strains formed biofilms and exhibited a strong biofilm phenotype in the presence and absence of antibiotics. However, addition of 40 *μ*g/mL riboflavin in combination with antibiotics reduced biofilm from strong to weak phenotype. Antibiofilm potential of riboflavin has been reported in *Staphylococcus aureus*, *E. coli*, *Candida albicans*, and *Enterococcus* spp. with reduction in biofilm formation following UV irradiation of riboflavin [23, 8]. In a similar study, combination of rifampicin and carbapenem showed activity against *Acinetobacter baumannii* biofilms [27].

Time‐kill curve analysis showed the bactericidal activity of antibiotic–riboflavin combinations, similar to rifampicin–carbapenem antibacterial activity [27]. These combinations, particularly meropenem displayed rapid bactericidal activity within 3–6 h. All the concentrations of riboflavin exhibited bactericidal activity with antibiotics; however, the highest was at 40–80 *μ*g/mL. Based on the observations, the antimicrobial effect of riboflavin in combination with antibiotics occurs within the first few hours of incubation, and concentrations of riboflavin above 20 *μ*g/mL provide enhanced synergistic bactericidal activity.

This study also presents a significant reduction in bacterial concentration for both *Salmonella* and *Shigella* strains within the macrophage cell lines. This reduction was dose‐dependent with riboflavin in the presence of the antibiotics tested. Significant clearance of bacterial strains from macrophages was between 12 and 24 h. Macrophages function to phagocytose, digest and destroy invading microorganisms such as bacteria. In a previous study, macrophage viability and activity were inhibited when riboflavin was deficient [28]. In addition, other studies reported that introduction of riboflavin induced mice resistance to bacterial infections by increasing production of neutrophils, macrophages and enhancing phagocytic activity of macrophages [8, 9, 29]. This study indicated that riboflavin potentiated fluoroquinolones and meropenem in a THP‐1 infection model, consistent with improved bacterial clearance.

It is important to emphasize that the synergistic concentration of 40 *μ*g/mL in our study is higher than the peak plasma concentrations (0.08–0.12 *μ*g/mL) achieved by oral administration [30]. Our findings are better contextualized as proof of concept where tested concentrations exceed plasma levels and delivery can only be through topical, local, or gastrointestinal routes. Direct translation based on systemic exposure alone is uncertain due to concentration differences. However, plasma concentration may not accurately reflect riboflavin exposure within the gut. Therefore, further studies are required to determine whether therapeutically local concentrations can be achieved.

## 5. Conclusions

The challenge of resistance requires the development of new therapeutic approaches in combating infections. In this study, riboflavin displayed a potentiating activity by synergistically enhancing the antimicrobial activity of fluoroquinolones and meropenem leading to reduction in resistant *Salmonella* and *Shigella* strains. Overall, under in vitro conditions, riboflavin enhanced antibiotic‐associated inhibition of MDR *Salmonella* and *Shigella*, most notably with meropenem relative to riboflavin‐only effects. These findings were obtained in four ICU‐derived isolates; hence, broader isolate panels and environmental variation are required for generalization. This in vitro resensitization of antibiotics could serve as therapeutic targets in mitigating resistance in selected bacterial pathogens.

## Author Contributions

Conceptualization: A.I.; methodology: A.I. and M.K.A.; validation: A.I.; formal analysis: A.I., M.K.A., E.A.A., E.W.B., and G.K.A.; investigation: A.I., M.K.A., E.A.A., E.W.B., and G.K.A.; resources: A.I.; data curation: A.I., M.K.A., E.A.A., E.W.B., and G.K.A.; writing—original draft preparation: A.I. and M.K.A.; writing—review and editing: A.I., M.K.A., E.A.A., E.W.B. and G.K.A.; visualization: A.I.; supervision: A.I.; project administration: A.I.; funding acquisition: A.I.

## Funding

This study was supported by ACE World Bank (ACE02‐WACCBIP: Awandare).

## Disclosure

All authors have read and agreed to the published version of the manuscript.

## Conflicts of Interest

The authors declare no conflicts of interest.

## Supporting Information

Additional supporting information can be found online in the Supporting Information section.

## Supporting information


**Supporting Information 1** Figure S1: Riboflavin and antibiotics combination against MDR *Salmonella* and *Shigella* species. (A) Effect of riboflavin and meropenem combination against multidrug‐resistant *Salmonella* and *Shigella* species. (B) Effect of riboflavin and levofloxacin combination against multidrug‐resistant *Salmonella* and *Shigella* species. (C) Effect of riboflavin and ciprofloxacin combination against multidrug‐resistant *Salmonella* and *Shigella* species. (D) Effect of riboflavin and ofloxacin combination against multidrug‐resistant *Salmonella* and *Shigella* species.

**Supporting Information 2** Figure S2: Time‐kill curve of selected antibiotics in combination with riboflavin at different concentrations. (A) Time‐kill curve of levofloxacin and riboflavin at different concentrations. (B) Time‐kill curve of ofloxacin and riboflavin at different concentrations. (C) Time‐kill curve of ciprofloxacin and riboflavin at different concentrations.

## Data Availability

All the data generated have been included in the manuscript and the supporting data.
