# Supplementary material for: An Exploratory Study on the Antibiotic Potentiation Activity of Riboflavin Against Biofilm‐Forming Multidrug‐Resistant Salmonella and Shigella Clinical Isolates
Source: Int J Microbiol. 2026 Oct 29;2026:1772416. doi: 10.1155/ijm/1772416 (PMC13720514; doi:10.1155/ijm/1772416)
Supplement: Supplementary file 1 [file IJM-2026-1772416-s001.docx]

**Supplementary I**

**Figure SIA**: Effect of riboflavin and meropenem combination against multidrug-resistant *Salmonella* and *Shigella* species

**Figure SIB**: Effect of riboflavin and levofloxacin combination against multidrug-resistant *Salmonella* and *Shigella* species

**Figure SIC**: Effect of riboflavin and ciprofloxacin combination against multidrug-resistant *Salmonella* and *Shigella* species

**Figure SID**: Effect of riboflavin and ofloxacin combination against multidrug-resistant *Salmonella* and *Shigella* species
