# Supplementary material for: An Exploratory Study on the Antibiotic Potentiation Activity of Riboflavin Against Biofilm‐Forming Multidrug‐Resistant Salmonella and Shigella Clinical Isolates
Source: Int J Microbiol. 2026 Oct 29;2026:1772416. doi: 10.1155/ijm/1772416 (PMC13720514; doi:10.1155/ijm/1772416)
Supplement: Supplementary file 2 [file IJM-2026-1772416-s002.docx]

**Supplementary II**

Time-kill curve of selected antibiotics in combination with riboflavin at different concentrations

**Figure SII:** Time-kill curve **(A)** Levofloxacin and riboflavin at different concentrations; (**B**) Ofloxacin and riboflavin at different concentrations (**C**) Ciprofloxacin and riboflavin at different concentrations
